# Hospital mortality among psychiatric inpatients: a cross-sectional study, Brazil, 2023

**DOI:** 10.1590/S2237-96222026v35e20250668.en

**Published:** 2026-02-09

**Authors:** Maximiliano Loiola Ponte de Souza

**Affiliations:** 1 Fundação Oswaldo Cruz, Fortaleza, CE, Brazil

**Keywords:** Mental Disorders, Hospital Mortality, Hospitals, Psychiatric, Health Inequality, Brazil, Trastornos Mentales, Mortalidad Hospitalaria, Hospitales Psiquiátricos, Desigualdades en Salud, Brasil

## Abstract

**Objectives:**

To estimate the hospital mortality rate and identify factors associated with death during psychiatric hospitalizations in Brazil in 2023.

**Methods:**

This is a cross-sectional retrospective study using data from the Brazilian Unified Health System Hospital Information System. All hospitalizations with primary diagnosis falling into codes F00-F99 of the 10th revision of the International Classification of Diseases were analyzed. The outcome was hospital death. Independent variables included sociodemographic, diagnostic and institutional characteristics. Multivariate binomial regression was used to estimate adjusted odds ratios (OR) and 95% confidence intervals (95%CI).

**Results:**

215,703 hospitalizations were analyzed, with 202 hospital deaths, resulting in a mortality rate of 0.9/1,000 admissions (95%CI 0.8; 1.1). Higher odds of death occurred among males (OR 1.61; 95%CI 1.17; 2.20), people aged 60+ years (OR 3.53; 95%CI 2.60; 4.80), White patients (OR 1.89; 95%CI 1.34; 2.67), in hospitalizations of up to 10 days (OR 4.16; 95%CI 3.10; 5.58) and in general hospitals (OR 1.51; 95%CI 1.12; 2.04). Lower odds were observed among patients >30 years old (OR 0.11; 95%CI 0.05; 0.24) and in hospitals under total municipal management (OR 0.74; 95%CI 0.55; 0.99).

**Conclusion:**

Despite the low hospital mortality rate, the results revealed inequalities and challenges in psychiatric care. Incorporating this indicator into mental health surveillance can contribute to improving management and safety of care within the Brazilian Unified Health System.

## Resumen

**Objetivos:**

Estimar la tasa de mortalidad hospitalaria e identificar los factores asociados con la muerte durante las hospitalizaciones psiquiátricas en Brasil en 2023.

**Métodos:**

Se realizó un estudio transversal retrospectivo utilizando datos del Sistema de Información Hospitalaria del Sistema Único de Salud. Se analizaron todas las hospitalizaciones con un diagnóstico principal entre los códigos F00 y F99 de la décima revisión de la Clasificación Internacional de Enfermedades. El desenlace fue la muerte hospitalaria. Las variables independientes incluyeron características sociodemográficas, diagnósticas e institucionales. Se utilizó regresión binomial multivariante para estimar las razones de momios ajustadas (*odds ratio*, OR) y los intervalos de confianza del 95 % (IC95%).

**Resultados:**

Se analizaron 215.703 hospitalizaciones, con 202 fallecimientos hospitalarios, lo que representa una tasa de 0,9 por cada 1.000 admisiones (IC95%: 0,8-1,1). La probabilidad de fallecimiento fue mayor en hombres (OR: 1,61; IC95%: 1,17-2,20), personas mayores de 60 años (OR: 3,53; IC95%: 2,60-4,80), pacientes de raza blanca (OR: 1,89; IC95%: 1,34-2,67), en hospitalizaciones de hasta 10 días (OR: 4,16; IC95%: 3,10-5,58) y en hospitales generales (OR: 1,51; IC95%: 1,12-2,04). Se observaron probabilidades más bajas entre los pacientes >30 años (OR 0,11; IC95% 0,05; 0,24) y en los hospitales bajo gestión municipal total (OR 0,74; IC95% 0,55; 0,99).

**Conclusión:**

A pesar de la baja tasa de mortalidad hospitalaria, los resultados revelaron desigualdades y desafíos en la atención psiquiátrica. La incorporación de este indicador a la vigilancia de la salud mental puede contribuir a mejorar la gestión y la seguridad de la atención dentro del Sistema Único de Salud de Brasil.

**Ethical aspects**

This research used public domain anonymized databases.

## Introduction

Hospitalization of people with mental disorders is indicated in contexts of acute exacerbation of their clinical picture, severe impairment of self-care, or imminent risk to the physical integrity of the individual in question or others (1). It is an extreme measure, with a therapeutic and protective purpose, aimed at preserving life in situations of intense vulnerability. 

Hospitalization can be associated with adverse events, including death during inpatient stay, with clinical, ethical and institutional repercussions. Thus, hospital mortality has been recognized as a critical indicator of the quality, safety and responsiveness of psychiatric services (2), and can be taken as a sentinel event, that is, a sign of a significant failure in health care, which requires analysis and immediate response from health service managers.

Available evidence indicates that hospital mortality rates in psychiatric settings tend to be relatively low when compared to those observed in general clinical specialties (3-5). However, when deaths do occur, they are often related to potentially preventable causes, such as undetected clinical conditions, delays in emergency care, or self-inflicted injuries during hospitalization (6-8). In low- and middle-income countries, these outcomes can be exacerbated by systemic limitations, including structural shortcomings in psychiatric hospitals and insufficient integration between mental health services and general medical care, especially among vulnerable populations (9,10).

Brazil, the largest and most populous country in South America, is marked by profound social and regional inequalities. Psychiatric hospitalizations in the country are regulated by the Brazilian Unified Health System (*Sistema Único de Saúde*, SUS), one of the largest public health systems with universal coverage in the world (11). In the last two decades, the SUS has prioritized deinstitutionalization policies and strengthening the psychosocial care network, with an emphasis on community services and care in freedom (12).

National guidelines now recommend prioritizing hospitalizations in general hospitals, with mental health beds integrated into the hospital network, although specialized public and contracted units with a predominantly psychiatric profile persist. The coexistence of diverse hospital facilities and community health services highlights the need to improve and monitor care at all points in the network, including in inpatient settings.

Given the magnitude of the population served by the SUS and the profound structural and social inequalities in Brazilian society, monitoring adverse events during mental health hospitalizations, including death, is fundamental for identifying service shortcomings and guiding improvements. Analysis of hospital mortality can play a relevant role in mental health surveillance by highlighting serious adverse events and pointing out weaknesses in the quality and safety of care provided. Furthermore, the production of evidence in this field, especially in low- and middle-income countries, can significantly contribute to the international debate on mental health reforms and the strengthening of public health systems.

The objectives of this study were to estimate the hospital mortality rate among patients hospitalized for mental and behavioral disorders in 2023 in Brazil and to identify associated variables.

## Methods

### Design 

This is a cross-sectional retrospective study with a quantitative and inferential approach, based on data from January to December 2023.

### Data sources

The data were obtained through the SUS Hospital Information System, run by the Ministry of Health. Classification of hospitals as psychiatric or general was carried out by linking the National Registry of Health Establishments code on the SUS Hospital Information System database, with corresponding information held on the National Registry of Health Establishments public database (https://cnes.datasus.gov.br/).

### Inclusion and exclusion criteria

Hospitalizations with primary diagnosis coded between F00 and F99 of the 10^th^ revision of the International Classification of Diseases (ICD-10) (13) were included in the study, with complete data for sex, age, race/skin color and municipality of residence. Records with missing or inconsistent information in any of these variables were excluded.

### Outcome of interest

The outcome was hospital death, considering only deaths that occurred in the same unit where hospitalization began. The hospital mortality rate was calculated as the number of hospital deaths divided by the total number of admissions in the period, multiplied by 1,000.

### Independent variables

The sociodemographic characteristics considered were: region of residence – South or Southeast (yes, no); sex (male, female); age <30 years (yes, no); age ≥60 years (yes, no); and race/skin color (White, non-White).

All subgroups were diagnosed based on ICD-10 and analyzed as binary variables (presence or absence), according to the main diagnostic category: F00-F09 (organic mental disorders); F10-F19 (mental and behavioral disorders due to psychoactive substance use); F20-F29 (schizophrenia, schizotypal and delusional disorders); F30-F39 (mood [affective] disorders); F40-F48 (neurotic, stress-related and somatoform disorders); F50-F59 (behavioral syndromes associated with psychological disturbances and physical factors); F60-F69 (disorders of adult personality and behavior); F70-F79 (mental retardation); F80-F89 (pervasive and specific developmental disorders); and F90-F99 (behavioral and emotional disorders with onset usually occurring in childhood and adolescence).

The health service characteristics were: length of inpatient stay ≤10 days (yes, no), as per previous studies (4,5,9); legal personality of the hospital (public, non-public); management of the hospital unit (total state management, total municipal management); and type of hospital (psychiatric, general).

### Sample

The sample included all eligible hospitalizations during the period analyzed, totaling 215,703 records.

### Statistical analysis

Descriptive statistics were used to characterize the study population. Bivariate associations between independent variables and hospital mortality were assessed using Pearson’s chi-square test. Variables with a p-value<0.20 in the bivariate analysis were included in the multivariate model.

Odds ratios (OR) and their respective 95% confidence intervals (95%CI) were estimated using binomial regression with a logit link function, a statistical technique equivalent to traditional logistic regression for binary outcomes. 

Data imputation was not performed. All analyses were conducted adopting a 5% significance level using Jamovi software (version 2.4.5.0).

## Results

We analyzed 215,703 hospitalizations for mental and behavioral disorders, recorded in 412 health facilities throughout Brazil. The majority of hospitalizations occurred in the South and Southeast regions of the country (66.6%), among male patients (63.2%), those over 30 years of age (72.7%), and non-White individuals (53.4%). The most prevalent diagnostic subgroups were F20-F29 (36.8%), F10-F19 (33.3%), and F30-F39 (21.5%). Most hospitalizations lasted more than 10 days (69.5%), occurred in non-public hospitals (65.3%), in hospitals under total municipal management (51.6%), and in psychiatric hospitals (72.1%) (Table 1). In all, 213 hospital deaths were recorded, which corresponded to a rate of 0.9 deaths/1,000 hospitalizations (95%CI 0.8; 1.1).

**Table 1:** Characteristics of mental and behavioral disorder hospitalizations. Brazil, 2023 (n=215,703)

Variable	n (%)
**Region of residence**	
South and Southeast	143,693 (66.6)
Other regions	72,010 (33.4)
Sex	
Male	136,337 (63.2)
Female	79,366 (36.8)
**Age group <30 years**	
Yes	58,837 (27.3)
No	156,866 (72.7)
**Age group ≥60 years**	
Yes	37,669 (17.5)
No	178,034 (82.5)
**Race/skin color**	
White	100,576 (46.6)
Non-White	115,127 (53.4)
**Diagnosis group (International Classification of Diseases – 10th revision – ICD-10)**	
F20-F29ᵃ	79,393 (36.8)
F10-F19ᵇ	71,900 (33.3)
F30-F39ᶜ	46,351 (21.5)
F00-F09ᵈ	5,651 (2.6)
F40-F48ᵉ	5,079 (2.4)
F60-F69ᶠ	3,271 (1.5)
F50-F59ᵍ	1,892 (0.9)
F70-F79ʰ	926 (0.4)
F80-F89ⁱ	544 (0.3)
F90-F99ʲ	696 (0.3)
**Length of inpatient stay**	
≤10 days	65,966 (30.6)
>10 days	149,737 (69.5)
**Legal personality of the hospital**	
Public	74,812 (34.7)
Non-public	140,891 (65.3)
**Type of hospital management**	
Total municipal management	111,354 (51.6)
Total state management	104,349 (48.4)
**Type of hospital**	
Psychiatric	155,453 (72.1)
General	60,250 (27.9)

^a^Schizophrenia, schizotypal and delusional disorders; ^b^Mental and behavioral disorders due to psychoactive substance use; ^c^Mood (affective) disorders; ^d^Organic mental disorders; ^e^Neurotic, stress-related and somatoform disorders; ^f^Disorders of adult personality and behavior; ^g^Behavioral syndromes associated with psychological disturbances and physical factors; ^h^Mental retardation; ^i^Pervasive and specific developmental disorders; ^j^Behavioral and emotional disorders with onset usually occurring in childhood and adolescence.

In the bivariate analysis (Table 2), statistically significant associations were identified between the outcome and the following variables: location in the South or Southeast regions (p-value<0.001), <30 years age group (p-value<0.001), ≥60 years age group (p-value<0.001), race/skin color (p-value<0.001), diagnosis codes F00-F09 (p-value<0.001), diagnosis codes F10-F19 (p-value 0.004), length of inpatient stay ≤10 days (p-value<0.001), type of hospital management (p-value<0.001), and type of hospital (p-value 0.017). The F20-F29 and F60-F69 variables and the legal personality of the hospital variable were included in the multivariate model because they presented a p-value<0.20.

**Table 2:** Odds ratios and 95% confidence intervals (95%CI) for hospital deaths, according to sociodemographic, clinical and hospitalization characteristics: bivariate analysis. Brazil, 2023 (n=215,703)

Variable	Deaths n (%)	Hospitalizations n (%)	Odds ratio (95%CI)	p-value
**Region of residence**				
South and Southeast	580 (0.4)	143,693 (66.6)	1.00	-
Other regions	508 (0.7)	72,010 (33.4)	1.76 (1.57; 1.97)	<0.001
Sex				
Male	864 (0.6)	136,337 (63.2)	1.00	-
Female	224 (0.3)	79,366 (36.8)	0.64 (0.55; 0.74)	<0.001
**Age group <30 years**				
Yes	69 (0.1)	58,837 (27.3)	0.22 (0.17; 0.28)	<0.001
No	1,019 (0.6)	156,866 (72.7)	1.00	-
**Age group ≥60 years**				
Yes	456 (1.2)	37,669 (17.5)	3.53 (3.10; 4.02)	<0.001
No	632 (0.4)	178,034 (82.5)	1.00	-
**Race/skin color**				
White	466 (0.5)	100,576 (46.6)	1.00	-
Non-White	622 (0.5)	115,127 (53.4)	1.10 (0.98; 1.24)	0.102
**Length of inpatient stay**				
≤10 days	292 (0.4)	65,966 (30.6)	1.00	-
>10 days	796 (0.5)	149,737 (69.5)	1.20 (1.04; 1.38)	0.014
**Type of hospital**				
Psychiatric	975 (0.6)	155,453 (72.1)	1.00	-
General	113 (0.2)	60,250 (27.9)	0.32 (0.26; 0.39)	<0.001

In the multiple binomial regression analysis (Table 3), higher odds of hospital death were observed among male patients (OR 1.61; 95%CI 1.17; 2.20), ≥60 years age group (OR 3.53; 95%CI 2.60; 4.80), White race/skin color (OR 1.89; 95%CI 1.34; 2.67), length of inpatient stay ≤10 days (OR 4.16; 95%CI 3.10; 5.58) and admission to a general hospital (OR 1.51; 95%CI 1.12; 2.04). The <30 years age group variable (OR 0.11; 95%CI 0.05; 0.24) and hospital under full municipal management variable (OR 0.74; 95%CI 0.55; 0.99) were associated with lower odds of death.

**Table 3:** Adjusted odds ratios and 95% confidence intervals (95%CI) for hospital deaths, according to sociodemographic, clinical and hospitalization characteristics: multiple analysis. Brazil, 2023 (n=215,703)

Variable	Adjusted odds ratio (95%CI)	p-value
**Region of residence**		
Other regions	1.48 (1.29; 1.70)	<0.001
Sex		
Female	0.66 (0.57; 0.78)	<0.001
**Age group <30 years**		
Yes	0.24 (0.18; 0.31)	<0.001
**Age group ≥60 years**		
Yes	2.96 (2.58; 3.39)	<0.001
**Race/skin color**		
Non-White	1.12 (0.99; 1.27)	0.068
**Length of inpatient stay**		
>10 days	1.16 (1.00; 1.33)	0.045
**Type of hospital**		
General	0.32 (0.26; 0.39)	<0.001

## Discussion

The hospital mortality rate among patients with mental and behavioral disorders was low, but this study revealed significant inequalities in specialized mental health care provided in hospital settings in Brazil. These findings reinforced the idea that hospital mortality in psychiatric contexts is a complex and multifactorial phenomenon, reflecting individual vulnerabilities and systemic failures in the provision of timely and adequate care. In this sense, this research suggested that measuring this indicator, combined with analysis of variables related to hospital death, can contribute to monitoring the quality and safety of mental health care within the SUS.

The hospital mortality rate observed in this study (0.9/1,000 admissions), on the one hand, was lower than that recorded in investigations with more specific populations, such as patients involuntarily hospitalized in Romania (5.13/1,000 admissions, 2000-2020) (7) and individuals with acute psychiatric conditions treated in a general hospital in Portugal (2.7/1,000 admissions, 1998-2013) (6). It was also below the rates described in African countries, such as Sudan (3.47/1,000 admissions, 2001-2009) (4) and Nigeria (12.9/1,000 admissions, 1976-1985) (9). On the other hand, it was similar to those found in Australia (1.12/1.000 admissions, 2002-2012) (8) and China (1.19/1,000 admissions, 2019-2020) (3). Brazil’s position as the most populous country in Latin America, coupled with the existence of a universal public health system, reinforces the relevance of these findings for global efforts to monitor mental health policies and services, with a view to overcoming health inequalities.

Males were associated with higher odds of hospital death, consistent with findings from various countries and historical contexts (3-9,15). This difference is widely documented and is usually attributed to a combination of factors, such as a high burden of cardiovascular disease, substance use, exposure to violence and underutilization of health services (16). When combined with social gender norms that discourage this population group from seeking help, these elements can contribute to the worsening of clinical conditions in psychiatric settings – especially where clinical care and mental health are not properly integrated (17).

Age was also associated with hospital mortality. Patients under 30 years old age had lower odds of death, while those 60 years of age or older had significantly higher odds (OR 3.53; p-value<0.001). This finding, consistent with previous studies (3,4,6,8), likely reflects a higher burden of comorbidities among the elderly and the limited capacity of psychiatric hospitals to diagnose and treat complex clinical conditions.

Not only in Brazil, but throughout Latin America, this issue takes on particular relevance given the rapidly aging population and the structural and financial weaknesses of hospital systems, which reduce the capacity to offer adequate care for elderly psychiatric patients (18-20). Similar challenges are seen in many middle-income countries. In order to address them, international guidelines recommend the implementation of geriatric protocols and the training of multidisciplinary teams, with the aim of reducing preventable deaths in aging psychiatric populations (21).

The variable most associated with hospital death was length of inpatient stay equal to or less than 10 days (OR 4.16; p-value<0.001). This suggests that many deaths occurred prematurely, possibly before the effective implementation of adequate clinical care. This pattern has been noted in low-income countries (Sudan, 2001-2009; India, 1983-2008; Nigeria, 1976-1985) (4,5,9) and middle-income countries (Romania, 2000-2020) (7), but not in studies conducted in high-income countries (Australia, 2002-2012) (8). This result indicates that the first days of hospitalization can be especially critical, particularly in contexts with scarce resources, fragmented care networks and low diagnostic capacity. In these scenarios, failures in initial clinical assessment and delays in reassessment can lead to admission of patients with acute clinical illnesses to psychiatric units, when referral to general hospitals would be more appropriate. These circumstances compromise the provision of timely and appropriate care (22).

Statistically significant association was found between lower odds of hospital death and hospitalizations in hospitals under total municipal management of the health system (OR 0.74; p-value 0.042). In the federative model of the SUS, this modality implies that the hospital is linked to a network coordinated by the municipality, with local responsibilities for the organization of care and tripartite agreement on resources. In contrast, hospitals under total state management remain under the direct responsibility of the state government, which may limit integration with other points in the health network (23).

The lower mortality rate observed in hospitals under total municipal management may reflect greater local capacity for coordination, management of care flows, and timely response in situations of clinical risk, especially for psychiatric hospitalizations. Even so, considering the borderline significance of the association and the complexity of the factors involved in hospital deaths, caution is recommended in interpreting this finding, the plausibility of which should be further investigated in future studies.

Two unexpected associations deserve attention. Although statistically robust, both require cautious interpretation and further investigation. The first refers to the higher odds of death among White patients (OR 1.86; p-value<0.001), which contradicts previous evidence indicating greater vulnerability of the non-White population to adverse health outcomes (24).

In this study, non-White patients accounted for the majority of hospitalizations. However, it is possible that a particularly vulnerable subgroup – non-White people with severe mental disorders and complex clinical comorbidities – face such significant barriers to access that they cannot even reach hospital services. As a consequence, these individuals would be at greater risk of death outside the system, which could veil the true magnitude of racial inequalities in mortality involving mental disorders. This pattern may be related to historical and persistent mechanisms of social exclusion, which operate structurally in Brazil and other countries in the region. Structural racism, widely recognized as a social determinant of health in Brazil and Latin America (24-26), limits access to specialized services and contributes to the invisibility of racialized groups, including in hospital care.

The second unexpected association relates to the higher odds of death in general hospitals compared to psychiatric hospitals, a result that contrasts with expectations, considering the history of precariousness of psychiatric institutions in Brazil (12). A possible explanation concerns patient profile: individuals with serious clinical comorbidities tend to be referred to general hospitals, expecting access to more comprehensive clinical care. However, these patients are frequently admitted to psychiatric wards or beds in these hospitals and, in many cases, remain under the exclusive responsibility of mental health teams, without effective access to specialized clinical services. Admission to a general hospital on its own does not guarantee integration between psychiatric and clinical care, a situation that is particularly critical in the case of patients with relevant clinical comorbidities. This care gap remains a structural challenge in Brazil and other middle-income countries, negatively impacting the safety and quality of hospital mental health care.

This study presented limitations inherent to cross-sectional analyses based on secondary data from public health information systems. The quality of records varied between facilities and regions, and lack of detailed clinical information limited the ability to assess individual risk and control for potential confounding factors. Our analysis only captured deaths that occurred in the same unit where hospitalization began, which made it impossible to monitor outcomes after transfers between institutions or after hospital discharge. This may have led to an underestimation of the true hospital mortality rate. Furthermore, the cross-sectional design prevented formulation of causal relationships between the variables analyzed and the outcomes observed. Despite these limitations, the inclusion of more than 200,000 hospitalizations and more than 200 deaths strengthened the analytical robustness of the study.

Based on the findings presented in this article, the potential of incorporating hospital mortality as an indicator for mental health surveillance was confirmed, insofar as it can identify care failures and guide actions aimed at improving the quality of care in psychiatric hospitalizations. As a sensitive indicator of the quality of hospital care and the organization of services, hospital mortality in psychiatric hospitalizations can be an important tool for guiding decisions and improving management in the SUS. Consolidation of this type of monitoring can also strengthen strategies for improving the quality of hospital care and contribute to the international debate on the quality and safety of psychiatric care, especially in low- and middle-income countries with structural challenges similar to the Brazilian case.

## Data Availability

The data used in this study are in the public domain and can be obtained by request from the Ministry of Health via its Mortality Information System, as previously described in the Methods section.
