# Supplementary material for: Hospital mortality among psychiatric inpatients: a cross-sectional study, Brazil, 2023
Source: Epidemiol Serv Saude. 2026 Feb 9;35:e20250668. doi: 10.1590/S2237-96222026v35e20250668.en (PMC12896745; doi:10.1590/S2237-96222026v35e20250668.en)
Supplement: Translation

# Translation: Mortalidade hospitalar em internações psiquiátricas: estudo transversal, Brasil, 2023

**DOI:** 10.1590/S2237-96222026v35e20250668.pt

**Authors:** Maximiliano Loiola Ponte de Souza

**Affiliations:** 1 Fundação Oswaldo Cruz, Fortaleza, CE, Brasil

**Keywords:** Transtornos Mentais, Mortalidade Hospitalar, Hospitais Psiquiátricos, Desigualdades em Saúde, Brasil

## Abstract

**Objetivos:**

Estimar a taxa de mortalidade hospitalar e identificar fatores associados ao óbito durante internações psiquiátricas no Brasil em 2023.

**Métodos:**

Trata-se de estudo transversal, retrospectivo, com dados do Sistema de Informações Hospitalares do Sistema Único de Saúde. Analisaram-se todas as internações com diagnóstico principal entre os códigos F00-F99 da 10ª revisão da Classificação Internacional de Doenças. O desfecho foi o óbito hospitalar. As variáveis independentes incluíram características sociodemográficas, diagnósticas e institucionais. Utilizou-se regressão binomial multivariada para estimar razões de chances (*odds ratio*, OR) ajustadas e intervalos de confiança de 95% (IC95%).

**Resultados:**

Foram analisadas 215.703 internações, com 202 óbitos hospitalares, resultando na taxa de 0,9/1 mil admissões (IC95% 0,8; 1,1). Maiores chances de óbito ocorreram entre homens (OR 1,61; IC95% 1,17; 2,20), pessoas com 60+ anos (OR 3,53; IC95% 2,60; 4,80), pacientes brancos (OR 1,89; IC95% 1,34; 2,67), em internações com até 10 dias (OR 4,16; IC95% 3,10; 5,58) e em hospitais gerais (OR 1,51; IC95% 1,12; 2,04). Menores chances foram observadas entre pacientes com >30 anos (OR 0,11; IC95% 0,05; 0,24) e em hospitais sob gestão plena municipal (OR 0,74; IC95% 0,55; 0,99).

**Conclusão:**

Apesar da baixa taxa de mortalidade hospitalar, os resultados revelaram desigualdades e desafios na atenção psiquiátrica. A incorporação desse indicador na vigilância em saúde mental pode contribuir para a qualificação da gestão e da segurança assistencial no Sistema Único de Saúde.

**Aspectos éticos**

Esta pesquisa utilizou bancos de dados de domínio público e anonimizados.

## Introdução

A internação hospitalar de pessoas com transtornos mentais é indicada em contextos de agudização do quadro clínico, de comprometimento grave do autocuidado ou de risco iminente à integridade física do próprio paciente ou de terceiros (1). Trata-se de recurso extremo, com finalidade terapêutica e protetiva, voltado à preservação da vida em situações de intensa vulnerabilidade. 

A hospitalização pode estar associada a eventos adversos, inclusive o óbito durante a internação, com repercussões clínicas, éticas e institucionais. Dessa forma, a mortalidade hospitalar tem sido reconhecida como indicador crítico da qualidade, da segurança e da capacidade de resposta dos serviços psiquiátricos (2), podendo ser tomada como um evento sentinela, isto é, um sinal de falha importante na atenção de saúde, que requer análise e resposta imediata por parte dos gestores.

As evidências disponíveis indicam que as taxas de mortalidade hospitalar em contextos psiquiátricos tendem a ser relativamente baixas ao serem comparadas às observadas em especialidades clínicas gerais (3-5). No entanto, quando os óbitos ocorrem, frequentemente estão relacionados a causas potencialmente evitáveis, como condições clínicas não detectadas, atrasos no atendimento emergencial ou lesões autoinfligidas durante a internação (6-8). Em países de baixa e de média renda, esses desfechos podem ser agravados por limitações sistêmicas, incluindo deficiências estruturais nos hospitais psiquiátricos e insuficiente integração entre os serviços de saúde mental e o cuidado médico geral, especialmente entre populações vulneráveis (9,10). 

O Brasil, maior e mais populoso país da América do Sul, é marcado por profundas desigualdades sociais e regionais. As internações psiquiátricas no país são reguladas pelo Sistema Único de Saúde (SUS), um dos maiores sistemas públicos de saúde com cobertura universal do mundo (11). Nas últimas duas décadas, o SUS tem priorizado políticas de desinstitucionalização e fortalecimento da rede de atenção psicossocial, com ênfase em serviços comunitários e em cuidados em liberdade (12). 

As diretrizes nacionais passaram a recomendar a realização preferencial das internações em hospitais gerais, com leitos de saúde mental integrados à rede hospitalar, embora persistam unidades especializadas, públicas e conveniadas com perfil predominantemente psiquiátrico. A coexistência entre os dispositivos hospitalares diversos e os serviços comunitários de saúde evidencia a necessidade de qualificar e de monitorar o cuidado em todos os pontos da rede, inclusive nos contextos de internação.

Dada a magnitude da população atendida pelo SUS e as profundas desigualdades estruturais e sociais da sociedade brasileira, o monitoramento de eventos danosos durante as internações em saúde mental, inclusive o óbito, é fundamental para identificar deficiências nos serviços e orientar melhorias. A análise da mortalidade hospitalar pode exercer papel relevante na vigilância em saúde mental, ao evidenciar eventos adversos graves e apontar fragilidades na qualidade e na segurança da atenção prestada. Além disso, a produção de evidências nesse campo, especialmente em países de baixa e de média renda, pode contribuir de forma significativa para o debate internacional sobre reformas em saúde mental e o fortalecimento de sistemas públicos de saúde.

Os objetivos deste estudo foram estimar a taxa de mortalidade hospitalar entre pacientes internados por transtornos mentais e do comportamento em 2023 no Brasil e identificar as variáveis associadas.

## Métodos

### Desenho do estudo 

Trata-se de estudo transversal, retrospectivo, com abordagem quantitativa e inferencial, com base em dados referentes ao período de janeiro a dezembro de 2023.

### Fontes de dados

Os dados foram obtidos através do Sistema de Informações Hospitalares do SUS, mantido pelo Ministério da Saúde. A classificação dos hospitais como psiquiátricos ou gerais foi realizada por meio da vinculação do código do Cadastro Nacional de Estabelecimentos de Saúde, presente no banco do Sistema de Informações Hospitalares do SUS, com informações da base pública do Cadastro Nacional de Estabelecimentos de Saúde online (https://cnes.datasus.gov.br/).

### Critérios de inclusão e exclusão

Foram incluídas no estudo as internações com diagnóstico principal codificado entre F00 e F99 da 10ª revisão da Classificação Internacional de Doenças (CID-10) (13), com dados completos para sexo, idade, raça/cor da pele e município de residência. Foram excluídos os registros com informações ausentes ou inconsistentes em qualquer uma dessas variáveis.

### Desfecho de interesse

O desfecho foi o óbito hospitalar, considerando-se apenas os óbitos ocorridos na mesma unidade onde a internação foi iniciada. A taxa de mortalidade hospitalar foi calculada como o número de óbitos hospitalares dividido pelo total de admissões no período, multiplicado por 1 mil.

### Variáveis independentes

As características sociodemográficas consideradas foram: região de residência – Sul ou Sudeste (sim, não); sexo (masculino, feminino), idade <30 anos (sim, não); idade ≥60 anos (sim, não); e raça/cor da pele (branca, não branca).

Todos os subgrupos foram diagnosticados com base na CID-10 e analisados como variáveis binárias (presença ou ausência), conforme a categoria principal de diagnóstico: F00-F09 (transtornos mentais orgânicos); F10-F19 (transtornos relacionados ao uso de substâncias psicoativas); F20-F29 (esquizofrenia, transtornos esquizotípicos e delirantes); F30-F39 (transtornos do humor [afetivos]); F40-F48 (transtornos neuróticos, relacionados ao estresse e somatoformes); F50-F59 (síndromes comportamentais associadas a disfunções fisiológicas e a fatores físicos); F60-F69 (transtornos da personalidade e do comportamento em adultos); F70-F79 (retardo mental); F80-F89 (transtornos do desenvolvimento psicológico); e F90-F99 (transtornos comportamentais e emocionais com início habitualmente na infância e na adolescência).

As características do serviço foram: tempo de internação ≤10 dias (sim, não), conforme estudos anteriores (4,5,9); natureza jurídica do hospital (público, não público); gestão da unidade hospitalar (plena estadual, plena municipal); e tipo de hospital (psiquiátrico, geral).

### Amostra

A amostra reuniu todas as internações elegíveis no período analisado, o que totalizou 215.703 registros.

### Análise estatística

Estatísticas descritivas foram utilizadas para caracterizar a população do estudo. As associações bivariadas entre as variáveis independentes e o óbito hospitalar foram avaliadas por meio do teste do qui-quadrado de Pearson. Variáveis com p-valor<0,20 na análise bivariada foram incluídas no modelo multivariado. 

As razões de chances (*odds ratio*, OR) e os respectivos intervalos de confiança de 95% (IC95%) foram estimados por meio de regressão binomial com função de ligação logito, técnica estatística equivalente à regressão logística tradicional para desfechos binários. 

A imputação de dados não foi realizada. Todas as análises foram conduzidas no software Jamovi (versão 2.4.5.0) e adotaram nível de significância de 5%.

## Resultados

Foram analisadas 215.703 internações por transtornos mentais e do comportamento, registradas em 412 estabelecimentos de saúde em todo o Brasil. A maioria das internações ocorreu nas regiões Sul e Sudeste (66,6%), entre pacientes do sexo masculino (63,2%), com idade superior a 30 anos (72,7%) e pessoas não brancas (53,4%). Os subgrupos diagnósticos mais prevalentes foram F20-F29 (36,8%), F10-F19 (33,3%) e F30-F39 (21,5%). A maioria das hospitalizações teve duração superior a 10 dias (69,5%), ocorreu em hospitais não públicos (65,3%), em hospitais sob gestão plena municipal (51,6%) e em hospitais psiquiátricos (72,1%) (Tabela 1). Foram registrados 213 óbitos hospitalares, o que correspondeu à taxa de 0,9 óbito/1 mil internações (IC95% 0,8; 1,1).

**Tabela 1:** Características das internações hospitalares por transtornos mentais e do comportamento. Brasil, 2023 (n=215.703)

Variável	n (%)
**Região de residência**	
Sul e Sudeste	143.693 (66,6)
Demais regiões	72.010 (33,4)
Sexo	
Masculino	136.337 (63,2)
Feminino	79.366 (36,8)
**Faixa etária <30 anos**	
Sim	58.837 (27,3)
Não	156.866 (72,7)
**Faixa etária ≥60 anos**	
Sim	37.669 (17,5)
Não	178.034 (82,5)
**Raça/cor da pele**	
Branca	100.576 (46,6)
Não branca	115.127 (53,4)
**Grupo diagnóstico (10a revisão da Classificação Internacional de Doenças – CID-10)**	
F20-F29ᵃ	79.393 (36,8)
F10-F19ᵇ	71.900 (33,3)
F30-F39ᶜ	46.351 (21,5)
F00-F09ᵈ	5.651 (2,6)
F40-F48ᵉ	5.079 (2,4)
F60-F69ᶠ	3.271 (1,5)
F50-F59ᵍ	1.892 (0,9)
F70-F79ʰ	926 (0,4)
F80-F89ⁱ	544 (0,3)
F90-F99ʲ	696 (0,3)
**Tempo de internação**	
≤10 dias	65.966 (30,6)
>10 dias	149.737 (69,5)
**Natureza jurídica do hospital**	
Público	74.812 (34,7)
Não público	140.891 (65,3)
**Tipo de gestão do hospital**	
Gestão plena municipal	111.354 (51,6)
Gestão plena estadual	104.349 (48,4)
**Tipo de hospital**	
Psiquiátrico	155.453 (72,1)
Geral	60.250 (27,9)

^a^Esquizofrenia, transtornos esquizotípicos e delirantes; ^b^Transtornos mentais e comportamentais devidos ao uso de substâncias psicoativas; ^c^Transtornos do humor (afetivos); ^d^Transtornos mentais orgânicos; ^e^Transtornos neuróticos, relacionados ao estresse e somatoformes; ^f^Transtornos da personalidade e do comportamento em adultos; ^g^Síndromes comportamentais associadas a disfunções fisiológicas e a fatores físicos; ^h^Retardo mental; ^i^Transtornos do desenvolvimento psicológico; ^j^Transtornos comportamentais e emocionais com início habitualmente na infância e adolescência.

Na análise bivariada (Tabela 2), foram identificadas associações estatisticamente significativas entre o desfecho e as seguintes variáveis: localização nas regiões Sul ou Sudeste (p-valor<0,001), faixa etária <30 anos (p-valor<0,001), faixa etária ≥60 anos (p-valor<0,001), raça/cor da pele (p-valor<0,001), diagnóstico F00-F09 (p-valor<0,001), diagnóstico F10-F19 (p-valor 0,004), tempo de internação ≤10 dias (p-valor<0,001), tipo de gestão do hospital (p-valor<0,001) e tipo de hospital (p-valor 0,017). As variáveis F20-F29 e F60-F69 e natureza jurídica do hospital foram incluídas no modelo multivariado por apresentarem p-valor<0,20.

**Tabela 2:** Razão de chances e intervalos de confiança de 95% (IC95%) para óbito hospitalar segundo características sociodemográficas, clínicas e de internação: análise bivariada. Brasil, 2023 (n=215.703)

Variável	Óbitos **n** (%)	Internações n (%)	Razão de chances (IC95%)	p-valor
**Região de residência**				
Sul e Sudeste	580 (0,4)	143.693 (66,6)	1,00	-
Demais regiões	508 (0,7)	72.010 (33,4)	1,76 (1,57; 1,97)	<0,001
Sexo				
Masculino	864 (0,6)	136.337 (63,2)	1,00	-
Feminino	224 (0,3)	79.366 (36,8)	0,64 (0,55; 0,74)	<0,001
**Faixa etária <30 anos**				
Sim	69 (0,1)	58.837 (27,3)	0,22 (0,17; 0,28)	<0,001
Não	1.019 (0,6)	156.866 (72,7)	1,00	-
**Faixa etária ≥60 anos**				
Sim	456 (1,2)	37.669 (17,5)	3,53 (3,10; 4,02)	<0,001
Não	632 (0,4)	178.034 (82,5)	1,00	-
**Raça/cor da pele**				
Branca	466 (0,5)	100.576 (46,6)	1,00	-
Não branca	622 (0,5)	115.127 (53,4)	1,10 (0,98; 1,24)	0,102
**Tempo de internação**				
≤10 dias	292 (0,4)	65.966 (30,6)	1,00	-
>10 dias	796 (0,5)	149.737 (69,5)	1,20 (1,04; 1,38)	0,014
**Tipo de hospital**				
Psiquiátrico	975 (0,6)	155.453 (72,1)	1,00	-
Geral	113 (0,2)	60.250 (27,9)	0,32 (0,26; 0,39)	<0,001

Na análise de regressão binomial múltipla (Tabela 3), observaram-se maiores chances de óbito hospitalar entre pacientes do sexo masculino (OR 1,61; IC95% 1,17; 2,20), faixa etária ≥60 anos (OR 3,53; IC95% 2,60; 4,80), raça/cor da pele branca (OR 1,89; IC95% 1,34; 2,67), tempo de internação ≤10 dias (OR 4,16; IC95% 3,10; 5,58) e admissão em hospital geral (OR 1,51; IC95% 1,12; 2,04). As variáveis faixa etária <30 anos (OR 0,11; IC95% 0,05; 0,24) e hospital sob gestão plena municipal (OR 0,74; IC95% 0,55; 0,99) estiveram associadas a menores chances de óbito.

**Tabela 3:** Razão de chances ajustada e intervalo de confiança de 95% (IC95%) para óbito hospitalar, segundo características sociodemográficas, clínicas e de internação: análise múltipla. Brasil, 2023 (n=215.703)

Variável	Razão de chances ajustada (IC95%)	p-valor
**Região de residência**		
Demais regiões	1,48 (1,29; 1,70)	<0,001
Sexo		
Feminino	0,66 (0,57; 0,78)	<0,001
**Faixa etária <30 anos**		
Sim	0,24 (0,18; 0,31)	<0,001
**Faixa etária ≥60 anos**		
Sim	2,96 (2,58; 3,39)	<0,001
**Raça/cor da pele**		
Não branca	1,12 (0,99; 1,27)	0,068
**Tempo de internação**		
>10 dias	1,16 (1,00; 1,33)	0,045
**Tipo de hospital**		
Geral	0,32 (0,26; 0,39)	<0,001

## Discussão

A taxa de mortalidade hospitalar entre pacientes com transtornos mentais e do comportamento foi baixa, mas este estudo revelou desigualdades significativas na atenção especializada em saúde mental prestada no contexto hospitalar no Brasil. Estes achados reforçaram que a mortalidade hospitalar em contextos psiquiátricos constitui um fenômeno complexo e multifatorial, o qual reflete vulnerabilidades individuais e falhas sistêmicas na oferta de cuidados oportunos e adequados. Nesse sentido, esta pesquisa sugeriu que a mensuração desse indicador, associada à análise das variáveis relacionadas ao óbito hospitalar, pode contribuir para o monitoramento da qualidade e da segurança da atenção em saúde mental no SUS.

A taxa de mortalidade hospitalar observada neste estudo (0,9/1 mil admissões), por um lado, foi inferior à registrada em investigações com populações mais específicas, como pacientes internados involuntariamente na Romênia (5,13/1 mil admissões, 2000-2020) (7) e indivíduos com quadros psiquiátricos agudos atendidos em um hospital geral em Portugal (2,7/1 mil admissões, 1998-2013) (6). Também se manteve abaixo das taxas descritas em países africanos, como Sudão (3,47/1 mil admissões, 2001-2009) (4) e Nigéria (12,9/1 mil admissões, 1976-1985) (9). Por outro lado, foi semelhante às encontradas na Austrália (1,12/1 mil admissões, 2002-2012) (8) e na China (1,19/1 mil admissões, 2019-2020) (3). A posição do Brasil como país mais populoso da América Latina, aliada à existência de um sistema público de saúde universal, reforça a relevância desses achados para os esforços globais de monitoramento das políticas e dos serviços de saúde mental, com vistas à superação de iniquidades em saúde.

O sexo masculino esteve associado a maiores chances de óbito hospitalar, em consonância com achados de diversos países e contextos históricos (3-9,15). Essa diferença é amplamente documentada e costuma ser atribuída a uma combinação de fatores, como elevada carga de doenças cardiovasculares, uso de substâncias, exposição à violência e subutilização dos serviços de saúde (16). Quando somados a normas sociais de gênero, que desestimulam esse estrato populacional da busca por ajuda, esses elementos podem contribuir para o agravamento de quadros clínicos em contextos psiquiátricos – especialmente onde a atenção clínica e a saúde mental não estão devidamente integradas (17).

A idade também apresentou associação com a mortalidade hospitalar. Pacientes com menos de 30 anos tiveram menores chances de óbito, enquanto aqueles com 60 anos ou mais apresentaram chances significativamente maiores (OR 3,53; p-valor<0,001). Esse achado, em consonância com estudos anteriores (3,4,6,8), reflete, provavelmente, maior carga de comorbidades entre idosos e limitada capacidade dos hospitais psiquiátricos em diagnosticar e tratar condições clínicas complexas. 

Não só no Brasil, mas em toda a América Latina, essa questão assume particular relevância diante do rápido envelhecimento populacional e das fragilidades estruturais e financeiras dos sistemas hospitalares, que reduzem a capacidade de oferecer cuidado adequado a pacientes psiquiátricos idosos (18-20). Desafios semelhantes são observados em muitos países de renda média. Para enfrentá-los, diretrizes internacionais recomendam a implementação de protocolos geriátricos e a capacitação de equipes multidisciplinares, com o objetivo de reduzir óbitos evitáveis em populações psiquiátricas envelhecidas (21).

A variável mais associada ao óbito hospitalar foi o tempo de internação igual ou inferior a 10 dias (OR 4,16; p-valor<0,001). Isso sugere que muitos óbitos ocorreram de forma precoce, possivelmente antes da implementação efetiva de cuidados clínicos adequados. Esse padrão tem sido notado em países de baixa renda (Sudão, 2001-2009; Índia, 1983-2008; Nigéria, 1976-1985) (4,5,9) e de média (Romenia, 2000-2020) (7), mas não nos conduzidos em países de alta renda (Austrália, 2002-2012) (8). O resultado indica que os primeiros dias de internação podem ser especialmente críticos, sobretudo em contextos com recursos escassos, redes de cuidado fragmentadas e baixa capacidade diagnóstica. Nesses cenários, falhas na avaliação clínica inicial e atrasos na reavaliação podem levar à admissão de pacientes com doenças clínicas agudas em unidades psiquiátricas, quando a referência a hospitais gerais seria mais apropriada. Essas circunstâncias comprometem a oferta de cuidado oportuno e adequado (22).

Foi observada associação estatisticamente significativa entre menores chances de óbito hospitalar e internações realizadas em hospitais sob gestão plena municipal do sistema de saúde (OR 0,74; p-valor 0,042). No modelo federativo do SUS, essa modalidade implica que o hospital está vinculado a uma rede coordenada pelo município, com responsabilidades locais sobre a organização da atenção e da pactuação tripartite de recursos. Em contrapartida, os hospitais sob gestão plena estadual permanecem sob responsabilidade direta do governo do estado, o que pode limitar a integração com os demais pontos da rede de saúde (23). 

A menor mortalidade observada em hospitais de gestão plena municipal pode refletir maior capacidade de articulação local, coordenação dos fluxos assistenciais e resposta oportuna em situações de risco clínico, especialmente em internações psiquiátricas. Ainda assim, considerando a significância limítrofe da associação e a complexidade dos fatores envolvidos nos óbitos hospitalares, recomenda-se cautela na interpretação desse achado, cuja plausibilidade deve ser aprofundada em estudos futuros.

Duas associações inesperadas merecem atenção. Embora estatisticamente robustas, ambas exigem interpretação cautelosa e investigações adicionais. A primeira refere-se à maior chance de óbito entre pacientes brancos (OR 1,86; p-valor<0,001), o que contraria evidências anteriores que indicam maior vulnerabilidade da população não branca a desfechos adversos em saúde (24). 

Neste estudo, pacientes não brancos concentraram a maior parte das internações. No entanto, é possível que um subgrupo particularmente vulnerável – pessoas não brancas com transtornos mentais graves e comorbidades clínicas complexas – enfrente barreiras de acesso tão significativas que sequer consiga alcançar os serviços hospitalares. Como consequência, esses indivíduos estariam sob maior risco de óbito fora do sistema, o que poderia invisibilizar a magnitude real das desigualdades raciais na mortalidade por transtornos mentais. Esse padrão pode estar relacionado a mecanismos históricos e persistentes de exclusão social, que operam de forma estrutural no Brasil e em outros países da região. O racismo estrutural, amplamente reconhecido como determinante social da saúde no Brasil e na América Latina (24-26), limita o acesso a serviços especializados e contribui para a invisibilidade de grupos racializados, inclusive na atenção hospitalar.

A segunda associação inesperada refere-se à maior chance de óbito em hospitais gerais, quando comparados a hospitais psiquiátricos, resultado contrastante com expectativas, considerando o histórico de precariedade das instituições psiquiátricas no Brasil (12). Uma explicação possível diz respeito ao perfil dos pacientes: indivíduos com comorbidades clínicas graves tendem a ser encaminhados para hospitais gerais, na expectativa de acesso a um cuidado clínico mais abrangente. No entanto, esses pacientes frequentemente são internados em enfermarias ou leitos psiquiátricos nesses hospitais e, em muitos casos, permanecem sob responsabilidade exclusiva das equipes de saúde mental, sem acesso efetivo aos serviços clínicos especializados. A simples admissão em um hospital geral não garante a integração entre os cuidados psiquiátricos e clínicos, situação particularmente crítica no caso de pacientes com comorbidades clínicas relevantes. Essa lacuna assistencial permanece como um desafio estrutural no Brasil e em outros países de renda média, impactando negativamente a segurança e a qualidade da atenção hospitalar em saúde mental.

Este estudo apresentou limitações inerentes às análises transversais baseadas em dados secundários provenientes de sistemas públicos de informação em saúde. A qualidade dos registros variou entre os estabelecimentos e as regiões, e a ausência de informações clínicas detalhadas limitou a capacidade de avaliar o risco individual e controlar potenciais fatores de confusão. A análise capturou apenas os óbitos ocorridos na mesma unidade em que a internação foi iniciada, o que impossibilitou o acompanhamento de desfechos após transferências entre instituições ou após evasão hospitalar. Isso pode ter levado à subestimação da verdadeira taxa de mortalidade hospitalar. Além disso, o delineamento transversal impediu a formulação de relações causais entre as variáveis analisadas e os desfechos observados. Apesar dessas limitações, a inclusão de mais de 200 mil internações e mais de 200 óbitos fortaleceu a robustez analítica do estudo.

A partir dos achados apresentados neste artigo, ratificou-se a potencialidade da incorporação da mortalidade hospitalar como indicador de vigilância em saúde mental, na medida em que ela pode identificar falhas assistenciais e orientar ações voltadas à qualificação do cuidado em internações psiquiátricas. Como indicador sensível à qualidade da atenção hospitalar e à organização dos serviços, a mortalidade hospitalar em internações psiquiátricas pode se constituir em um instrumento importante para orientar decisões e aprimorar a gestão no SUS. A consolidação desse tipo de monitoramento pode ainda fortalecer estratégias de qualificação da atenção hospitalar e contribuir para o debate internacional sobre qualidade e segurança da atenção psiquiátrica, especialmente em países de baixa e de média renda, com desafios estruturais semelhantes ao caso brasileiro.

## Data Availability

Os dados utilizados neste estudo são de domínio público e podem ser obtidos mediante solicitação ao Ministério da Saúde, por meio do Sistema de Informações sobre Mortalidade, conforme descrito previamente na seção Métodos.
